# Supplementary material for: Rhizobia Contribute to Salinity Tolerance in Common Beans (Phaseolus vulgaris L.)
Source: Cells. 2022 Nov 16;11(22):3628. doi: 10.3390/cells11223628 (PMC9688157; doi:10.3390/cells11223628)
Supplement: Supplementary file 1 [file cells-11-03628-s001.zip › cells-2012761-Table S1.pdf]

Table S1: Primers used in qPCR

| **Gene name** | **Primer name** | **Primer sequence** |
|---|---|---|
| Glutathione synthase | GluA_F | ATGGCCTACATCACCTCGAC |
| | GluA_R | GGAAAATTCGGTGACGAAGA |
| Glutamate decarboxylase 1 | GAD1_F | AGCCAGCCTTCTCACTGTTTTC |
| | GAD1_R | TGATCGCGTTCACCAAAGCAAG |
| ABC transporter permease | AMC88_RS22265_F | GCCTTCATGTCGCTGATCTT |
| | AMC88_RS22265_R | CCGTCAGGCTCTCATAGCTC |
| potassium-transporting ATPase | AMC88_RS24375_F | GGGCATCCTTCCGTATAACC |
| | AMC88_RS24375_R | GCCATCTGGGTGAGATAGGA |
| aspartate aminotransferase | AMC88_RS29380_F | CGGCACGACAAGAAGAAGAT |
| | AMC88_RS29380_R | CGAAGGCATTGTGGAAGAGT |
| gyrase B | gyrB_F | CCAGATGCCGGAGCTGATCG |
| | gyrB_R | CTGATCGATGTGCCGGGCAT |
| ATP synthase (ATPase) | aptA_F | CCTACCGCCAGATGTCGCTG |
| | atpA_R | AGGCCCTGTTCGAACTTGCC |
