# Supplementary material for: Rhizobia Contribute to Salinity Tolerance in Common Beans (Phaseolus vulgaris L.)
Source: Cells. 2022 Nov 16;11(22):3628. doi: 10.3390/cells11223628 (PMC9688157; doi:10.3390/cells11223628)
Supplement: Supplementary file 1 [file cells-11-03628-s001.zip › cells-2012761-Table S3.pdf]

Table S3: Biological pathway analysis of upregulated bacteroid genes when host common bean plants were exposed to 300 mM NaCl.

==> Amino acids transport and metabolisms <==

COG0014 Gamma-glutamyl phosphate reductase 1
COG0031 Cysteine synthase 1
COG0075 Serine-pyruvate aminotransferase/archaeal aspartate aminotransferase 2
COG0079 Histidinol-phosphate/aromatic aminotransferase and cobyric acid decarboxylase 2
COG0160 4-aminobutyrate aminotransferase and related aminotransferases 1
COG0403 Glycine cleavage system protein P (pyridoxal-binding), N-terminal domain 1
COG0404 Glycine cleavage system T protein (aminomethyltransferase) 1
COG0410 ABC-type branched-chain amino acid transport systems, ATPase component 12
COG0411 ABC-type branched-chain amino acid transport systems, ATPase component 12
COG0436 Aspartate/tyrosine/aromatic aminotransferase 2
COG0444 ABC-type dipeptide/oligopeptide/nickel transport system, ATPase component 12
COG0458 Carbamoylphosphate synthase large subunit (split gene in MJ) 2
COG0460 Homoserine dehydrogenase 1
COG0473 Isocitrate/isopropylmalate dehydrogenase 1
COG0477 Permeases of the major facilitator superfamily 1
COG0498 Threonine synthase 1
COG0509 Glycine cleavage system H protein (lipoate-binding) 1
COG0559 Branched-chain amino acid ABC-type transport system, permease components 1
COG0601 ABC-type dipeptide/oligopeptide/nickel transport systems, permease components 1
COG0626 Cystathionine beta-lyases/cystathionine gamma-synthases 3
COG0665 Glycine/D-amino acid oxidases (deaminating) 4
COG0683 ABC-type branched-chain amino acid transport systems, periplasmic component 1
COG0687 Spermidine/putrescine-binding periplasmic protein 1
COG0697 Permeases of the drug/metabolite transporter (DMT) superfamily 1
COG0703 Shikimate kinase 1
COG0747 ABC-type dipeptide transport system, periplasmic component 1
COG0765 ABC-type amino acid transport system, permease component 2
COG0834 ABC-type amino acid transport/signal transduction systems, periplasmic component/domain 2
COG1045 Serine acetyltransferase 1
COG1063 Threonine dehydrogenase and related Zn-dependent dehydrogenases 1
COG1104 Cysteine sulfinate desulfinase/cysteine desulfurase and related enzymes 1
COG1124 ABC-type dipeptide/oligopeptide/nickel transport system, ATPase component 12
COG1125 ABC-type proline/glycine betaine transport systems, ATPase components 11
COG1126 ABC-type polar amino acid transport system, ATPase component 12
COG1167 Transcriptional regulators containing a DNA-binding HTH domain and an aminotransferase domain (MocR family) and their eukaryotic orthologs 2
COG1168 Bifunctional PLP-dependent enzyme with beta-cystathionase and maltose regulon repressor activities 1
COG1171 Threonine dehydratase 1
COG1173 ABC-type dipeptide/oligopeptide/nickel transport systems, permease components 2
COG1174 ABC-type proline/glycine betaine transport systems, permease component 2
COG1176 ABC-type spermidine/putrescine transport system, permease component I 2
COG1177 ABC-type spermidine/putrescine transport system, permease component II 2
COG1231 Monoamine oxidase 2
COG1748 Saccharopine dehydrogenase and related proteins 1
COG1897 Homoserine trans-succinylase 1
COG1921 Selenocysteine synthase [seryl-tRNASer selenium transferase] 2
COG1984 Allophanate hydrolase subunit 2 1
COG2008 Threonine aldolase 1
COG2171 Tetrahydrodipicolinate N-succinyltransferase 1
COG2303 Choline dehydrogenase and related flavoproteins 2
COG2873 O-acetylhomoserine sulfhydrylase 2
COG3075 Anaerobic glycerol-3-phosphate dehydrogenase 1
COG3185 4-hydroxyphenylpyruvate dioxygenase and related hemolysins 1
COG3842 ABC-type spermidine/putrescine transport systems, ATPase components 11

COG3844 Kynureninase 1
COG3977 Alanine-alpha-ketoisovalerate (or valine-pyruvate) aminotransferase 1
COG4160 ABC-type arginine/histidine transport system, permease component 1
COG4161 ABC-type arginine transport system, ATPase component 11
COG4166 ABC-type oligopeptide transport system, periplasmic component 1
COG4175 ABC-type proline/glycine betaine transport system, ATPase component 12
COG4176 ABC-type proline/glycine betaine transport system, permease component 2
COG4177 ABC-type branched-chain amino acid transport system, permease component 1
COG4215 ABC-type arginine transport system, permease component 1
COG4597 ABC-type amino acid transport system, permease component 1
COG4598 ABC-type histidine transport system, ATPase component 11
COG4608 ABC-type oligopeptide transport system, ATPase component 12
COG4992 Ornithine/acetylornithine aminotransferase 1

==> Inorganic ion transport and metabolism <==
COG0444 ABC-type dipeptide/oligopeptide/nickel transport system, ATPase component 12
COG0474 Cation transport ATPase 1
COG0477 Permeases of the major facilitator superfamily 1
COG0573 ABC-type phosphate transport system, permease component 1
COG0581 ABC-type phosphate transport system, permease component 1
COG0600 ABC-type nitrate/sulfonate/bicarbonate transport system, permease component 3
COG0601 ABC-type dipeptide/oligopeptide/nickel transport systems, permease components 1
COG0609 ABC-type Fe3+-siderophore transport system, permease component 1
COG0715 ABC-type nitrate/sulfonate/bicarbonate transport systems, periplasmic components 2
COG1116 ABC-type nitrate/sulfonate/bicarbonate transport system, ATPase component 11
COG1117 ABC-type phosphate transport system, ATPase component 11
COG1118 ABC-type sulfate/molybdate transport systems, ATPase component 11
COG1119 ABC-type molybdenum transport system, ATPase component/photorepair protein PhrA 12
COG1120 ABC-type cobalamin/Fe3+-siderophores transport systems, ATPase components 11
COG1121 ABC-type Mn/Zn transport systems, ATPase component 12
COG1122 ABC-type cobalt transport system, ATPase component 11
COG1124 ABC-type dipeptide/oligopeptide/nickel transport system, ATPase component 12
COG1135 ABC-type metal ion transport system, ATPase component 12
COG1173 ABC-type dipeptide/oligopeptide/nickel transport systems, permease components 2
COG1178 ABC-type Fe3+ transport system, permease component 5
COG1785 Alkaline phosphatase 1
COG2011 ABC-type metal ion transport system, permease component 2
COG2060 K+-transporting ATPase, A chain 1
COG2072 Predicted flavoprotein involved in K+ transport 2
COG2216 High-affinity K+ transport system, ATPase chain B 1
COG2217 Cation transport ATPase 1
COG2223 Nitrate/nitrite transporter 1
COG2382 Enterochelin esterase and related enzymes 1
COG3221 ABC-type phosphate/phosphonate transport system, periplasmic component 1
COG3420 Nitrous oxidase accessory protein 1
COG3454 Metal-dependent hydrolase involved in phosphonate metabolism 2
COG3624 Uncharacterized enzyme of phosphonate metabolism 1
COG3638 ABC-type phosphate/phosphonate transport system, ATPase component 12
COG3639 ABC-type phosphate/phosphonate transport system, permease component 2
COG3696 Putative silver efflux pump 4
COG3703 Uncharacterized protein involved in cation transport 1
COG4100 Cystathionine beta-lyase family protein involved in aluminum resistance 1
COG4107 ABC-type phosphonate transport system, ATPase component 12
COG4148 ABC-type molybdate transport system, ATPase component 12
COG4149 ABC-type molybdate transport system, permease component 2

COG4521 ABC-type taurine transport system, periplasmic component 1
COG4525 ABC-type taurine transport system, ATPase component 11
COG4555 ABC-type Na+ transport system, ATPase component 12
COG4559 ABC-type hemin transport system, ATPase component 12
COG4604 ABC-type enterochelin transport system, ATPase component 11
COG4605 ABC-type enterochelin transport system, permease component 1
COG4606 ABC-type enterochelin transport system, permease component 1
COG4615 ABC-type siderophore export system, fused ATPase and permease components 10
COG4778 ABC-type phosphonate transport system, ATPase component 11
COG4779 ABC-type enterobactin transport system, permease component 1
COG4986 ABC-type anion transport system, duplicated permease component 4

==> Carbohydrate transport and metabolism <==
COG0063 Predicted sugar kinase 1
COG0448 ADP-glucose pyrophosphorylase 1
COG0451 Nucleoside-diphosphate-sugar epimerases 4
COG0477 Permeases of the major facilitator superfamily 1
COG0697 Permeases of the drug/metabolite transporter (DMT) superfamily 1
COG0702 Predicted nucleoside-diphosphate-sugar epimerases 2
COG1070 Sugar (pentulose and hexulose) kinases 1
COG1082 Sugar phosphate isomerases/epimerases 1
COG1086 Predicted nucleoside-diphosphate sugar epimerases 2
COG1129 ABC-type sugar transport system, ATPase component 18
COG1134 ABC-type polysaccharide/polyol phosphate transport system, ATPase component 12
COG1172 Ribose/xylose/arabinose/galactoside ABC-type transport systems, permease components 1
COG1175 ABC-type sugar transport systems, permease components 2
COG1653 ABC-type sugar transport system, periplasmic component 1
COG1820 N-acetylglucosamine-6-phosphate deacetylase 3
COG1879 ABC-type sugar transport system, periplasmic component 1
COG1940 Transcriptional regulator/sugar kinase 2
COG2182 Maltose-binding periplasmic proteins/domains 1
COG2211 Na+/melibiose symporter and related transporters 1
COG2271 Sugar phosphate permease 1
COG2301 Citrate lyase beta subunit 1
COG2814 Arabinose efflux permease 2
COG2956 Predicted N-acetylglucosaminyl transferase 1
COG3090 TRAP-type C4-dicarboxylate transport system, small permease component 1
COG3525 N-acetyl-beta-hexosaminidase 1
COG3622 Hydroxypyruvate isomerase 1
COG3839 ABC-type sugar transport systems, ATPase components 11
COG4209 ABC-type polysaccharide transport system, permease component 1
